# Role of ultrasonographic features and quantified *BRAF*V600E mutation in lymph node metastasis in Chinese patients with papillary thyroid carcinoma

**DOI:** 10.1038/s41598-018-36171-z

**Published:** 2019-01-11

**Authors:** Liang Guo, Ya-qi Ma, Yao Yao, Meng Wu, Zi-hui Deng, Feng-wei Zhu, Yu-kun Luo, Jie Tang

**Affiliations:** 1 Departments of Ultrasound, Chinese People’s Liberation Army General Hospital, Beijing, People’s Republic of China; 2 Departments of Pathology, Chinese People’s Liberation Army General Hospital, Beijing, People’s Republic of China; 3 Institute of Geriatrics, Chinese People’s Liberation Army General Hospital, Beijing, People’s Republic of China; 4 Research Laboratory of Biochemistry, Basic Medical Institute, Chinese People’s Liberation Army General Hospital, Beijing, People’s Republic of China

## Abstract

The association between cervical lymph node metastasis (LNM) and ultrasonographic features as well as *BRAF*^V600E^ mutations in patients with papillary thyroid carcinoma (PTC) remained controversial. This study investigated the association between LNM and ultrasonographic features as well as *BRAF*^V600E^ mutation in Chinese patients with PTC. A total of 280 patients with PTC in China were included in this study. 108 had cervical lymph node metastasis, while 172 had not. Younger age (<45years) and several ultrasonographic features were significantly associated with cervical LNM (*P*s < 0.05). The *BRAF*^V600E^ mutation was detected in 81.0% of patients with PTC (226/280). The status of *BRAF*^V600E^ mutation was not associated with cervical LNM. However, Ct values by PCR and intensity of reactions by immunohistochemistry (IHC) for *BRAF*^V600E^ expression had shown significant difference between group with and without LNM. Furthermore, an increased proportion of LNM was also found with the incremental intensity of IHC for *BRAF*^V600E^ expression from weak to strong reaction after adjusted potential confounders. Further studies are required to verify this association and explore the intrinsic mechanism.

## Introduction

Papillary thyroid carcinoma (PTC) is the most common thyroid cancer, accounting for 80–90% of all thyroid carcinomas, with an increasing incidence globally^1,2^. PTC cancer cells metastases primarily via cervical lymph node metastases (LNM). The clinical significance of LNM in PTC remained controversial. Previous studies reported that LNM may influence only recurrence but not survival^3^. However, recent evidence from a large-scale nested case-control study indicated that LNM and incomplete surgical excision were two primary characteristics associated with higher morbidity^4^, and the presence of LNM enhanced the rate of distant metastases by 11.2-fold^5^. Cervical ultrasonography (US) is commonly performed as preoperative imaging to visualize the LNMs lesions. However, factors including lymph nodes size and air or bone shadowing might limit their detection by US, thus influence the preoperative strategy.

*BRAF* somatic mutations are the commonest genetic alterations in PTC, which may have diagnostic, prognostic, and therapeutic value in the management of PTC. *BRAF*^V600E^ mutation resulting from the substitution of a valine by a glutamate at reside 600, is associated with adverse prognostic factors, such as extrathyroidal extension, LN metastasis, and poor survival^6,7^, because this mutation, via activation of MAP kinase pathway, can cause loss of expression of thyroid genes and refractoriness to radioiodine, as well as up-regulation of angiogenic and tumor-promoting molecules^8,9^. However, the definite prognostic role of the *BRAF* mutation^10,11^ was not identified in every studies. A recent large Chinese cohort study revealed that the association of this mutation with extrathyroidal invasion and LNM were not seen in all cities when analyzed city by city^12^. Hence, in our Chinese population with PTC, the relationship between *BRAF*^V600E^ mutation and cervical LNM cannot be concluded.

In this study, we aim to identified certain US features of primary tumor which might be able to predict LNM in PTC patients, and evaluate the *BRAF*^V600E^ mutation from US-FNA in a qualitative manner with immunohistochemical staining and PCR in order to investigate the association between this mutation and cervical LNM in Chinese PTC patients, a population in which the research on the quantification of this mutated allele and its protein expression remains understudied, at the background of the large variation in the prevalence of the *BRAF*^V600E^ mutations in PTCs among countries.

## Results

### General characteristics of patients with PTC according to the status of LNM

Among the 280 patients, the mean age ± SD of the patients was 43.78 ± 10.93 years (range, 9–73years) and postoperative pathological results showed that 108 PTCs had cervical LNM, while 172 PTCs had not (Table 1). PTCs with LNM were younger than PTCs without LNM (40.48 ± 11.83 *vs*. 45.85 ± 9.81, *p* < 0.001), and there were more male patients suffering from LNM than females (55.4% *vs*. 33.5%, *p* = 0.001). In terms of personal habits (smoking and alcohol intake) and ethnicity, there were no significant differences between the two groups (*p* > 0.05).

**Table 1:** General characteristics of our PTC patients and *BRAF*^V600E^ mutation in PTC patients with and without LNM.

Characteristics		Metastasis no. (%)		*X*^2^/*t* value	*P* value
		Yes (n = 108)	No (n = 172)		
Age (years)		40.48 ± 11.83	45.85 ± 9.81	*t* = −4.117	<0.001
	<45 y	65 (46.4)	75 (53.6)	*X*^2^ = 7.295	0.007
	≥45 y	43 (30.7)	97 (69.3)		
Gender	Male	36 (55.4)	29 (44.6)	*X*^2^ = 10.099	0.001
	Female	72 (33.5)	143 (66.5)		
Ethnic	Han	100 (38.2)	162 (61.8)	*X*^2^ = 0.280	0.597
	Others	8 (44.4)	10 (55.6)		
Smoke	Yes	19 (50)	19 (50)	*X*^2^ = 2.424	0.119
	No	89 (36.8)	153 (63.2)		
Alcohol	Yes	17 (48.6)	18 (51.4)	*X*^2^ = 1.688	0.194
	No	91 (37.1)	154 (62.9)		

### The US characteristics and *BRAF*^V600E^ status with its Ct values

**Figure 1:**
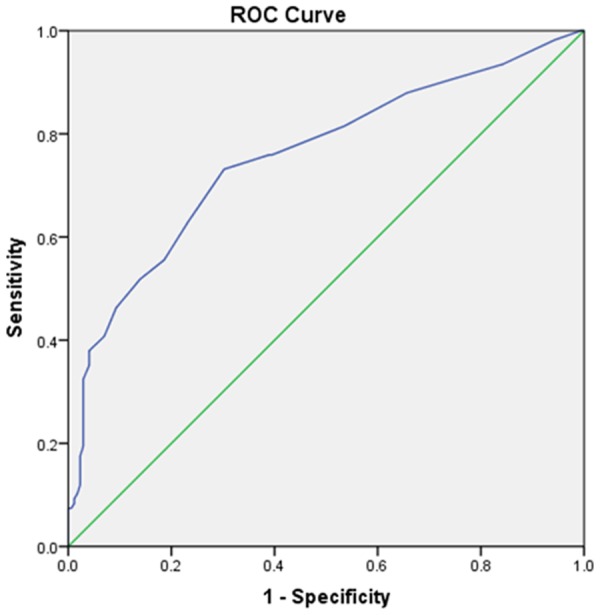
Receiver-operating Characteristic (ROC) curve of nodule size for predicting the risk of cervical LNM. Blue line shows an ROC plot for nodule size with an AUC of 0.76 (95% CI: 0.695–0.816).

The mean nodule maximum diameter was 1.10 ± 0.68 (range, 0.30–4.5 cm). The mean maximum diameters of PTCs with LNM was larger than those without LNM (1.46 ± 0.85 cm *vs*. 0.87 ± 0.40 cm, *p* < 0.001) **(**Table 2**)**. The area under curve (AUC) to distinguish cervical LNM from none cervical LNM was 0.76 (95%CI: 0.695–0.816)for tumor size with cut-off value of 0.95 cm with an AUC of 0.76 according to the ROC curves (sensitivity, 73.1%; specificity, 69.8%) (Fig. 1). There were significant differences in other sonographic features between PTC patients with and without LNM, including multifocality, shape, margin, heterogeneous echogenicity, calcification, CDFI, US-LNM, distance to capsule and the diffuse disease (*P*s < 0.05). However, the status of *BRAF*^V600E^ showed no significant difference between two groups (p = 0.499).

**Table 2:** Ultrasonographic characteristics and *BRAF*^V600E^ status of PTC patients with and without cervical LNM.

Parameters	Characteristics	Metastasis no. (%)		*X*^2^/*t*/*U* value	*P value*
		Yes (n = 108)	No (n = 172)		
Multifocality	Yes	40 (49.4)	41 (50.6)	*X*^2^ = 5.622	0.018
	No (Single)	68 (34.2)	131 (65.8)		
Size, cm		1.46 ± 0.85	0.87 ± 0.40	*t* = 7.835	<0.001
Shape	Regular	23 (25.0)	69 (75.0)	*X*^2^ = 10.651	0.001
	Irregular	85 (45.2)	103 (54.8)		
Margin	Well-defined	24 (29.3)	58 (70.7)	*X*^2^ = 4.236	0.040
	Ill-defined	84 (42.4)	114 (57.6)		
Echogenicity	Homogeneous	81 (33.5)	161 (66.5)	*X*^2^ = 19.577	<0.001
	Heterogeneous	27 (71.0)	11 (29.0)		
	Hypoechoic	101 (38.4)	162 (61.6)	*X*^2^ = 0.560	0.972
	Marked-hypoechoic	5 (41.7)	7 (58.3)		
	Hyper/iso-echoic	2 (40)	3 (60)		
Calcification	Absent	23 (23.5)	75 (76.5)	*X*^2^ = 14.896	0.001
	Macro-calcification	3 (60)	2 (40.0)		
	Micro-calcification	82 (46.3)	95 (53.7)		
CDFI	Rich	31 (77.5)	9 (22.5)	*X*^2^ = 29.847	<0.001
	Not rich	77 (32.1)	163 (67.9)		
US-LNM	Yes	69 (94.5)	4 (5.5)	*X*^2^ = 130.455	<0.001
	No	39 (18.8)	168 (81.2)		
Taller than wide	>1	68 (39.3)	105 (60.7)	*X*^2^ = 0.103	0.748
	≤1	40 (37.4)	67 (62.6)		
Distance to capsule	≤2 mm	100 (49.0)	104 (51)	*X*^2^ = 34.627	<0.001
	>2 mm	8 (10.5)	68 (89.5)		
Diffuse disease	Yes	16 (14.8)	45 (26.2)	*X*^2^ = 5.014	0.025
	No	92 (85.2)	127 (73.8)		
*BRAF*^V600E^ mutation	Yes	85 (37.6)	141 (62.4)	*X*^2^ = 0.457	0.499
	No	23 (42.6)	31 (57.4)

### General information and ultrasonographic indicators of cervical LNM in PTC patients

**Figure 2:**
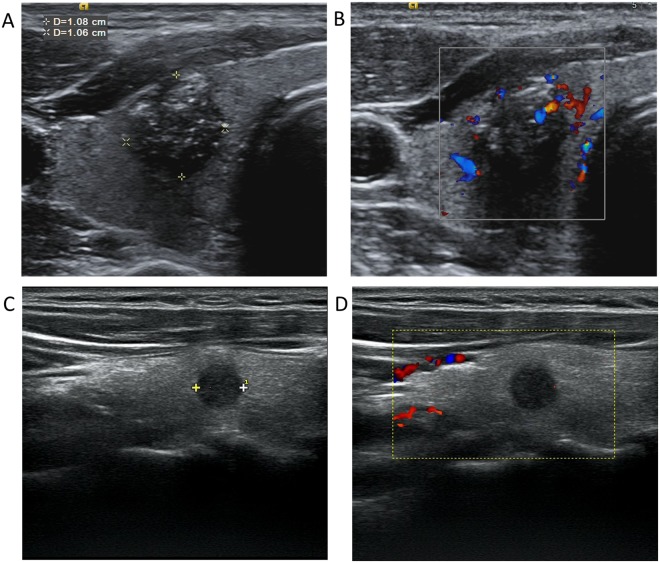
Ultrasonographic images of a PTC patient with (**A**,**B**) and without (**C**,**D**) cervical lymph node metastasis. (**A**) A nodule with certain malignant signs, such as hypoechogenicity, irregular shape, ill-defined margin, multiple microcalcification and capsule invasion; (**B**) CDFI showed rich blood flow in and around the nodule (**A**). (**C**) A hypoechoic nodule in the middle of the right thyroid, smaller than that in (**A**), with regular shape, defined margin, and no signs of capsule invasion; (**D**) CDFI showed lack of the blood flow signals, compared with that in (**B**). CDFI: Color Doppler Flow Imaging.

The multivariate logistic regression revealed significant associations of ages < 45 years (OR = 2.446; 95%CI: 0.880–3.695), multifocality (OR = 2.113; 95%CI: 1.070–4.170:), tumor size (OR = 3.565; 95%CI: 1.784–7.123), CDFI (OR = 3.783; 95%CI: 1.348–10.613) and close to capsule (OR = 4.181; 95%CI: 1.770–9.877) with cervical LNM (Fig. 2) (Table 3). However, *BRAF*^V600E^ mutation (OR = 1.082; 95%CI: 0.503–2.327) was not significantly associated.

**Table 3:** Multivariate analysis of the association between clinicopathological characteristics and cervical LNM in 280 PTC patients.

Characteristics	Metastases no. (%)		Multivariate analysis	
	Yes	No	OR (95% CI)	*p* value
Total number	108	172		
Male	36 (33.3)	29 (16.9)	1.803 (0.880–3.695)	0.108
Age < 45 y	65 (60.2)	75 (43.6)	2.446 (1.314–4.551)	0.005
Multifocality (yes)	40 (37.0)	41 (23.8)	2.113 (1.070–4.170)	0.031
Tumor size > 0.95 cm	79 (73.1)	52 (30.2)	3.565 (1.784–7.123)	<0.001
Margin (Ill-defined)	84 (77.8)	114 (66.3)	1.473 (0.676–3.207)	0.330
Shape (Irregular)	85 (78.7)	103 (59.9)	1.511 (0.730–3.128)	0.266
Microcalcification	82 (75.9)	95 (55.2)	1.525 (0.794–2.930)	0.205
CDFI (Rich)	31 (28.7)	9 (5.2)	3.783 (1.348–10.613)	0.011
Distance to capsule (<2 mm)	100 (92.6)	104 (60.5)	4.181 (1.770–9.877)	0.001
Diffuse disease	16 (14.8)	45 (26.2)	0.653 (0.302–1.409)	0.277
*BRAF*^V600E^ mutation positive	85 (78.7)	141 (82.0)	1.082 (0.503–2.327)	0.840

### *BRAF*^V600E^ Ct value with PCR and its expression by IHC staining in *BRAF*^V600E^-positive PTC patients with and without cervical LNM

**Figure 3:**
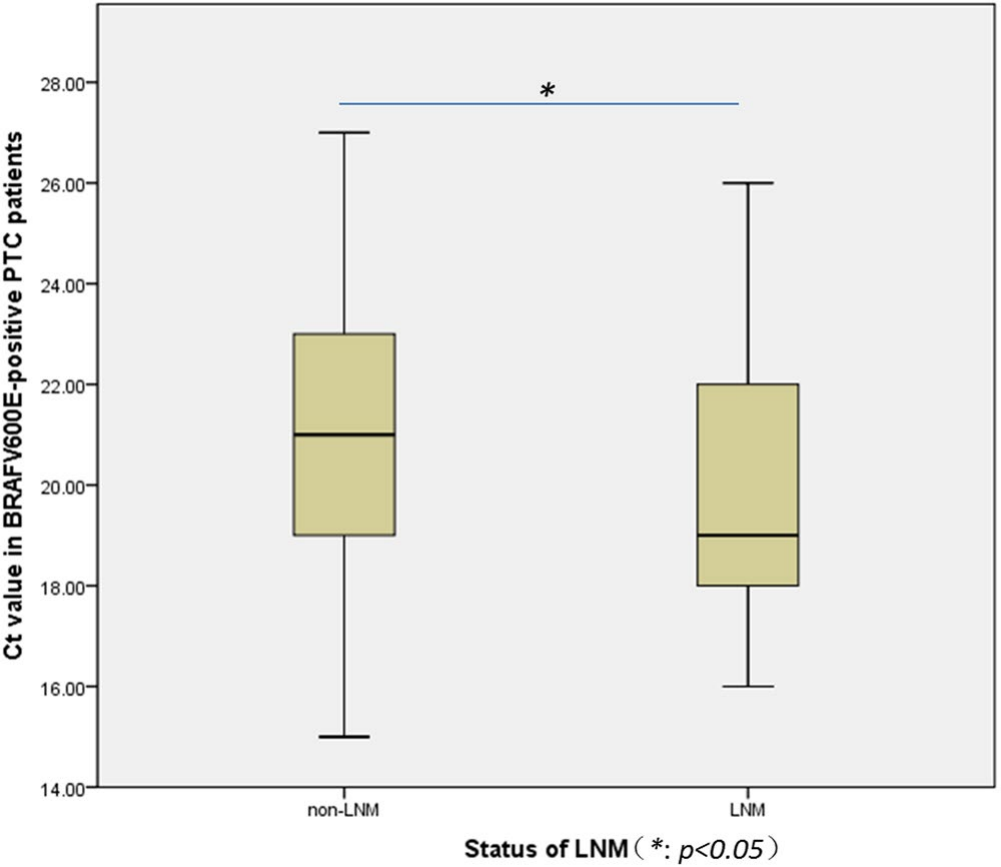
Box plot of Ct values in BRAFV600E-positive PTC patients grouped by the status of LNM. There was significant difference of Ct values expressed by median between groups with (Ct value = 19) and without LNM (Ct value = 21).

**Figure 4:**
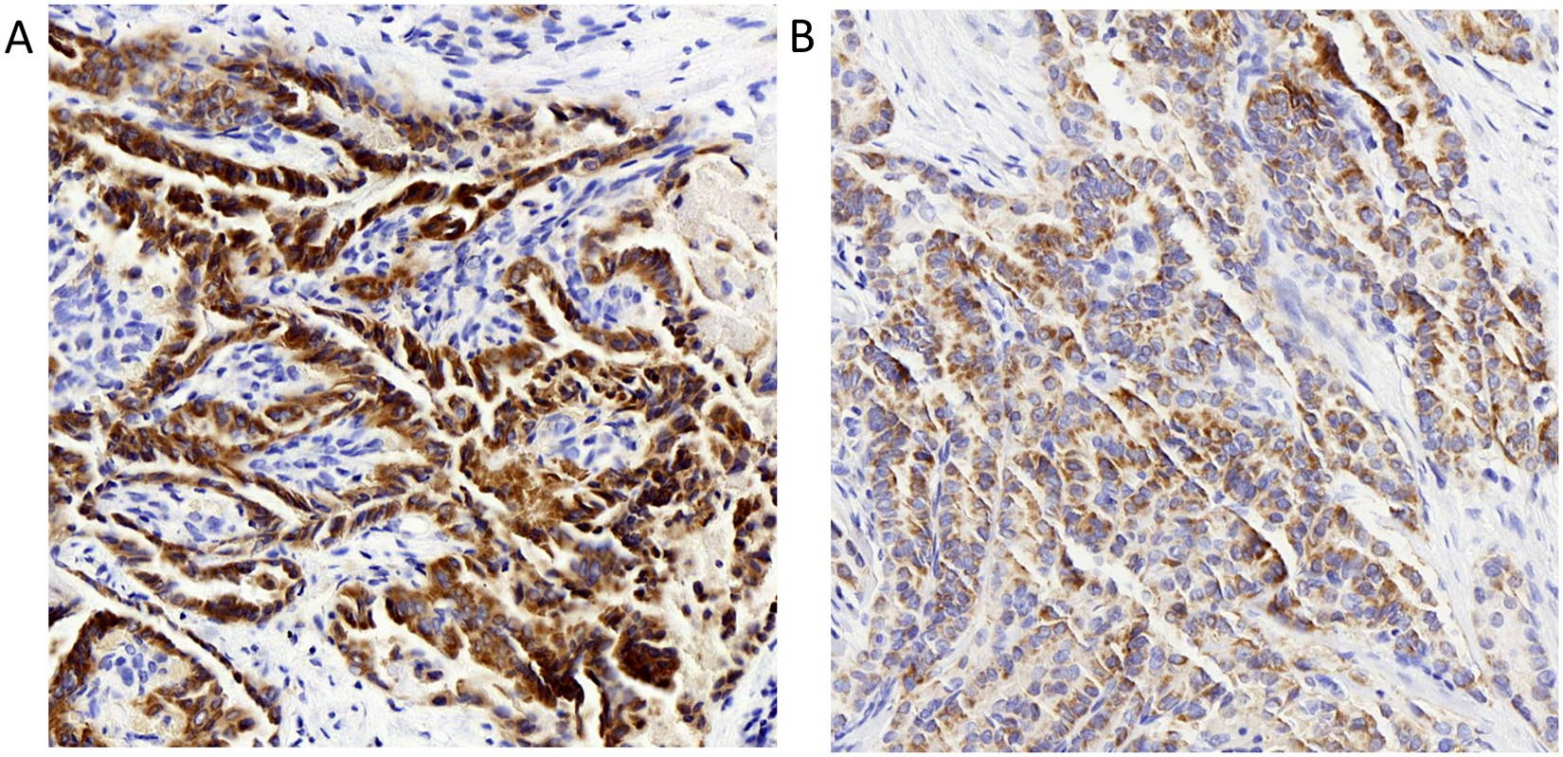
*BRAF*^V600E^ IHC staining in *BRAF*^V600E^-positive (by PCR) PTC with (**A**) and without (**B**) lymph node metastasis. Representative staining of the PTC nodules with *BRAF*^V600E^ specific antibody (VE1), as shown by the brown staining (magnification, ×200). (**A**) Strong staining (+++); (**B**) Weak staining (+). IHC: immunohistochemical.

Among the *BRAF*^V600E^-positive patients (226/280), the *BRAF*^V600E^ Ct value was significantly lower in LNM group (*p* = 0.002) (Fig. 3) **(**Table 4**)**. Furthermore, the *BRAF*^V600E^ protein expression by IHC showed that patients with cervical LNM had a higher percentage of the strong reaction than those without LNM (62.4% *vs*. 35.5%, *p* < 0.001) (Fig. 4).

**Table 4:** *BRAF*^V600E^ Ct value by PCR and its expression by IHC staining in BRAFV600E-positive PTC patients with and without cervical LNM.

		Metastasis (*BRAF*^V600E^+) No. (%)		*U* value	*p* value
		Yes (n = 85)	No (n = 141)		
Ct value of *BRAF*^V600E^ mutation^*^		19	21	*U* = 4511.000	0.002
Intensity of IHC for *BRAF*^V600E^ expression	+	8 (9.4)	42 (29.8)	*U* = 7912.000	<0.001
	++	24 (28.2)	49 (34.8)		
	+++	53 (62.4)	50 (35.5)		

^*^BRAF^V600E^ Ct values are expressed as median.

### Multivariate logistic analysis for determining the association of LNM with different level of *BRAF*^V600E^ expression by Ct value and IHC intensity

Odds ratios for the LNM in non-mutation group (Ct value ≥ 28) and relatively low *BRAF*^V600E^ expression group (Ct value 22–27) versus high *BRAF*^V600E^ expression group (Ct value ≤ 19) were 0.86 (*p* > 0.05) and 0.42 (*p* = 0.012), respectively; while after fully adjusted by covariates in model 3, no significant association was found (Table 5). However, as far as the level of *BRAF*^V600E^ expression by IHC intensity was concerned, the odds ratios for weak and moderate reaction group were 0.18 (95%CI: 0.08–0.42) and 0.46 (95%CI: 0.25–0.86), respectively, as compared to the strong reaction group. After adjusted for several potential confounders in model 3, the association of LNM and *BRAF*^V600E^ expression grouped by IHC intensity remained significant. Furthermore, an increased proportion of LNM was also found with the incremental intensity of IHC for *BRAF*^V600E^ expression from weak to strong reaction, with an increased odds ratio from 0.38 (95%CI: 0.15–0.96) to 0.70 (95%CI: 0.33–1.47) as compared to the strong reaction group.

**Table 5:** Odds Ratios and 95% confidence intervals for LNM according to the different groups of *BRAF*^V600E^ Ct value and intensity of IHC for *BRAF*^V600E^ expression.

	Ct value of *BRAF*^V600E^ mutation	OR	95% CI	*p*	Intensity of IHC for *BRAF*^V600E^ expression	OR	95% CI	*p*
Model 1	≥28	0.86	0.43–1.73	0.676	IHC negative	0.70	0.36–1.36	0.292
	22–27	0.42	0.22–0.83	0.012	IHC weak	0.18	0.08–0.42	<0.001
	21–20	0.76	0.40–1.45	0.399	IHC moderate	0.46	0.25–0.86	0.015
	≤19	1.00	—		IHC strong	1.00	—	
Model 2	≥28	0.97	0.47–2.00	0.935	IHC negative	0.77	0.38–1.53	0.453
	22–27	0.50	0.25–1.00	0.051	IHC weak	0.21	0.09–0.51	0.001
	21–20	0.83	0.42–1.62	0.586	IHC moderate	0.50	0.26–0.95	0.035
	≤19	1.00	—		IHC strong	1.00	—	
Model 3	≥28	0.99	0.41–2.35	0.975	IHC negative	0.82	0.37–1.81	0.627
	22–27	0.98	0.42–2.30	0.961	IHC weak	0.38	0.15–0.96	0.042
	21–20	0.96	0.43–2.13	0.912	IHC moderate	0.70	0.33–1.47	0.346
	≤19	1.00	—		IHC strong	1.00	—	

Model 1 was unadjusted; Model 2 was adjusted for sex, age, ethnicity, smoking and alcohol drinking habits; Model 3 was further adjusted multifocality, Tumor size, margin, shape, microcalcification, CDFI, distance to capsule, and diffuse disease based on Model 2.

## Discussion

Among all the general characteristics, only younger age (<45years) was an independent predictor to LNM, which is consistent with previous reports that younger age was associated with an increased risk of cervical LNM in PTC. As Ning *et al*. suggested, younger age may indicate greater risk for the evolution of biological aggressiveness; hence appropriate initial management may improve the prognosis of younger PTC patients^13^.

Due to the high specificity and positive predictive value, ultrasonography is an important tool for the detection of metastatic nodes, however, only half of the LNM that are found during surgery can be identified by preoperative US, because US evaluation is an operator-dependent technique^14^ and unable to consistently visualize deep anatomic structures, or structures that are acoustically shadowed by bone or air.

Nam *et al*. demonstrated that PTCs with malignant US features had worse biological behaviors than PTCs without, including extrathyroidal extension, LNM, and advanced stage^15^. In other words, US features during diagnosis can serve as a useful predictive tool for biological behavior in PTC. In this study, some US features of the primary tumor showed significant differences between LNM group and non-LNM group. These features were multifocality, tumor size, shape, margin, calcification, CDFI and the distance to the capsule. However after multivariate logistic regression analysis, only multifocality, tumor size, CDFI and distance to capsule were significantly associated with LNM.

Multifocality was associated with higher probability of disease recurrence and poorer prognosis as compared to unifocal disease^16^. In close proximity to the previous finding^17^, our results showed that 29% of patients had multifocal PTC. As suggested by Kim *et al*., the number of tumor foci independently predicted LNM^18^. The clonal origin of the multifocal PTCs has not been completely identified to date. It is not clear whether these foci represent intraglandular dissemination of a single primary tumor or arise from distinct progenitor cells.

With the increase of tumor’s diameter, the infiltration depth and scope becomes deeper and wider, respectively, resulting in increased contact areas of tumor with thyroid capsule and intraglandular lymphovascular, which might increase the incidence rate of cervical LNM. Sezer *et al*. suggested that the lymphovascular invasion was significantly associated with an increased risk of cervical lymph node metastasis (OR = 30.61;95CI:14.99–62.49)^19^. Our results also showed that the optimal cut-off value of the tumor size for predicting the risk of cervical LNM was 0.95 cm, in proximity to 1 cm which is one of the diagnostic criteria of PTMC^20^. Most of tumors this size are indolent low risk tumors, but some of them behave more aggressively^21^. Therefore, as suggested by Nam-Goong *et al*., small tumor size alone does not assure low risk in incidentally identified thyroid cancers^22^.

Liu *et al*. indicated that only capsule invasion and tumor location were significantly associated with cervical LNM^23^. In this study, we found that when the primary tumor was close (<2 mm) or even clung to the thyroid capsule (the latter often exhibited a blurred boundary between the tumor and capsule, suggesting the breach of the continuity of the capsule, which is a sign of capsule invasion demonstrated by postoperative pathology), the patients were predisposed to cervical LNM. It is well established that the growth of thyroid cancer through a tissue barrier can reflect the invasive properties of the thyroid primary tumor, which continues to form an integral element of tumor staging systems^24^.

Angiogenesis is one of the important factors in tumor growth, metastasis and progression^25^. It is also a precursor for regional LNM^26^ and reflects microvessel density in local tumor progression^27^. CDFI has potential to detect blood flow differences of healthy organs and cancerous tissues, which can be used to reflect the the status of microvessels in PTC^28^. Some studies found that PTCs with high VEGF-A expression, which is one of the major regulators of tumor angiogenesis have a significantly higher microvessel density. This is associated with an increased risk of recurrence and worse prognosis^29,30^. Schluter *et al*. explained that the microenvironment of the primary tumor characterized by highly expressed angiogenic biomarkers prepared the tumor for metastasis, but some metastases were clinically detectable at far later time points^25^.

BRAF mutation that was first identified in malignant melanoma by Davies and colleagues in 2002^31^ is the most prevalent type of genetic alteration in thyroid cancer and has been widely investigated. The incidence rate of *BRAF*^V600E^ varies greatly, ranging from 29–83% in PTC, the reason for which is unclear, although it is suggested that geographic, genetic factors, or other factors may account for this^32^. Our results showed that 81% (226/280) PTCs harbored *BRAF*^V600E^ mutation, which is a higher incidence rate within the range mentioned above.

Besides its strong correlation with PTC, *BRAF*^V600E^ mutation is found to associate with aggressive behavior and poor prognosis that are defined by extrathyroidal extension, multicentricity, local recurrence, LNM, and distant metastasis^33^. Xing^8^ evaluate pooled prognostic data from all published studies and found a significant association between the BRAF mutation and LNM (OR: 1.83; 95% CI: 1.58–2.13) of PTC. However, other studies did not demonstrate this association. Kathleen *et al*. indicated that *BRAF*^V600E^ mutation was not found to be significantly associated with the presence of LNM (P = 0.167) and multivariate analysis showed only size and venous/lymphatic invasion were significantly associated with LNM^34^. Consistent with this finding, our results showed no positive association between the status of the *BRAF*^V600E^ mutation and the cervical LNM, and no difference between the two groups with and without LNM. The conflicting results of these studies might be due to variations in the study populations in terms of size, age distribution, histological variants, genetic factors, environmental factors, disease stages at the time of initial diagnosis, and methods or criteria used to detect the *BRAF*^V600E^ mutation^35,36^.

Ct value was inversely related to the *BRAF*^V600E^ mRNA level. Thus, a low Ct value corresponded to a higher mRNA level^37^. In this article, our findings showed that Ct values were lower in patients with LNM, which were in line with another retrospective study by Vivian *et al*.^38^. However, their study population was mainly Korean, with Ct40 as the cut-off value, whereas our diagnostic criterion for *BRAF*^V600E^ mutation was Ct28. Immunohistochemistry was utilized to evaluate the level of *BRAF*^V600E^ protein. Coinciding with the difference of Ct values in two groups, strong reactions were mainly found in the PTC patients with LNM. However, when logistic regression was performed, especially in model 3 adjusted for several potential confounders, only IHC for *BRAF*^V600E^ expression grouped by intensity from weak to strong reaction had shown an increased proportion of LNM. There may be several reasons for this inconsistent role of Ct value and IHC in LNM. First, Ct value quantitatively reflects gene expression in real-time PCR, but it is determined from a log-linear plot of the PCR signal versus the cycle number, thus it is not a linear term^37^. Second, mRNA is unstable and easy to be degraded. Third, the procedure is regulated by many factors during the translation of mRNA to protein, which plays the ultimate role in the biological function. Fourth there may be sampling errors in FNA due to the heterogeneous distribution of *BRAF*^V600E^ mutation within the tumors^39^.

Therefore, some PTCs with a higher expression of *BRAF*^V600E^ protein may represent a proclivity to aggressive pathologic features as compared to those of lower expressions. In a vitro study, *BRAF*^V600E^-overexpressed rat thyroid cells that were grown on Matrigel^TM^ showed increase in migration of thyroid cells^40^; *in vivo*, the percentage of mutant BRAF alleles was positively associated with tumor burden and extrathyroidal invasion in PTC^41^. Therefore, it may not be enough to test only the status of *BRAF*^V600E^ from FNA cells; quantification of this mutated gene is required, especially for the patients who have the US signs mentioned above.

There are four limitations to our study. First, a selection bias is inevitable due to patients exclusion whose thyroid nodules were suspected of malignance examined, but without receiving further cytopathologic diagnosis or undergoing operation in our hospital. Second, this is a retrospective study, in which some images were not real-time reviewed; therefore, these results may be different if the attending surgeons performed the US. Third, diagnostic performance varied in accordance to the methodology of *BRAF*^V600E^ testing and Ct cut-off values; therefore, the results may not be reproducible if another method and cut-off value are used. Finally, the study population was composed of Chinese patients, who have a higher prevalence of BRAF mutation; therefore, the conclusion of this study may not represent the situations in other countries, especially in areas with a low prevalence of BRAF mutation.

In conclusion, certain ultrasonographic features that are the sign of malignancy during examination of thyroid nodules, were associated with cervical LNM. Furthermore, we suggested a quantification of the *BRAF*^V600E^ as a supplement for just testing the status of the mutated gene from FNA cells in *BRAF*^V600E^-positive patients. Therefore, the combination of US features and the quantification of the *BRAF*^V600E^ could serve as an effective tool for risk stratification and determination of the initial surgical approach in PTC patients preoperatively.

## Methods

### Patients and specimens

This retrospective observational study was approved by Chinese People’s Liberation Army General Hospital (PLA General Hospital) Research Ethics Committees. We confirmed that all methods were performed in accordance with the relevant guidelines and regulations. Written informed consent was obtained from all patients for US-guided fine-needle aspiration (US-FNA) and *BRAF*^V600E^ mutation prior to each procedure. We included 280 PTC patients who underwent surgery at PLA General Hospital between 2016 and 2017. Exclusion criteria were: (1) Patients who refused US-FNA before thyroidectomy or *BRAF*^V600E^ mutation analysis of surgical specimen. (2) Patients who did not have preoperative US in our hospital or whose lesion was unable to be identified on US. (3) Patients who underwent thyroid nodule minimally invasive ablation, any cervical surgery, chemical therapy or radiotherapy. (4) Patients with benign tumor or other types of thyroid carcinoma.

The general characteristics of the 280 patients were collected, including age, sex, ethnicity, and lifestyles (smoking and alcohol drinking habits). According to previous reports, 45years was set as the cut-off value; <45years was defined as younger age.

All patients had either total thyroidectomy or near-total thyroidectomy, and received prophylactic or therapeutic central-compartment neck dissection. Lateral compartmental lymph node dissection was performed for patients with US-FNA-proven or clinically suspicious lateral cervical lymphadenopathy. Fresh PTC specimens were collected from these 280 patients undergoing thyroidectomy at PLA General Hospital. Immunohistochemistry was performed after formalin fixed and paraffin-embedded PTC tumor specimens from these patients were collected. Two experienced pathologists were delegated to review histopathological slides retrospectively for all cases to confirm the histological diagnosis.

### Ultrasound examination

US imaging was performed by radiologists with 5–10 years of experience with Philips iU22 system (Philips, Amsterdam, Holland) equipped with a L12–5 linear probe at the frequency of 9–12 MHz. US images were obtained from all patients; thyroidnodules were analyzed according to the following sonographic features: nodular size, internal component (solid or cystic), echogenicity in respect to the thyroid parenchyma and strap muscle (hyperechogenicity, isoechogenicity, hypoechgenicity or marked hypoechogenicity), margin characteristics (well defined or ill defined), shape (taller than wide or wider than tall), and presence/absence of microcalcifications. The largest lesion with maximum diameter was analyzed when more than 3 nodules suspicious of malignancies were detected in the thyroid. The internal component was classified as solid in case of thyroid nodules with over 50% solid component. Echogenicity was classified as compared with the adjacent thyroid gland. US findings with at least one of these features: taller-than-wide shape, ill-defined margins, over 50% solid component, marked hypoechogenicity and the presence of microcalcifications are indicative of malignancy.

### *BRAF*^V600E^ mutation analysis

The BRAFV600E mutation analysis was performed with DNA that extracted from remaining FNA cells after cytologic evaluation. Real-time PCR was performed using the CFX96 real-time PCR detection system (Bio-Rad, Hercules, CA, USA). The mutant BRAF gene (encoding BRAF V600E) was amplified with specific primers. Thermal cycling conditions were initial denaturation of 1 cycle for 5 minutes at 95 °C, 95 °C for 10 minutes (1 cycle), and 95 °C for 15 seconds, followed by 15 cycles of 95 °C for 25 s, 64 °C for 20 s, and 72 °C for 20 s with a final step of annealing and elongation of 31cycles at 93 °C for 25 s, 60 °C for 35 s and 72 °C for 20 s. The BRAF V600E mutation status of each primary PTC was determined using the AmoyDx BRAF V600E Mutation Detection Kit (Amoy Diagnostics). The FAM signals of the mutation detection system indicate the mutation status of the sample. The HEX/VIC signals indicate the internal control status. The FAM Ct value was checked for each sample: a) If the sample FAM Ct value ≥28, the sample was classified as negative or below the detection limit of the kit. b) If the sample FAM Ct value < 28, the sample was classified as mutation positive.

### Immunohistochemistry

Histological sections were fixed with 4% formalin, embedded in paraffin, cut and mounted on glass slides, stained with hematoxylin and eosin. Immunohistochemical analyses were made using the *BRAF*^V600E^ mutation-specific antibody (VE1, ZM-0302, 1:30, Zhongshan Jinqiao Biological Technology Co., Ltd.). The paraffin-embedded tissue blocks were cut in 4um sections. mounted on coated glass slides and held in a drying oven at 60 °C for 2 h. Immunohistochemistry (IHC) was performed using the EnVision FLEX+ (DK-2600; Dako Denmark A/S, Glostrup, Denmark).The IHC staining results were evaluated independently by 2 observers who were blinded to all genetic and clinical data. When clear cytoplasmic staining with VE1 antibody was observed, the result was interpreted as positive and scored as weak (+), moderate (++), or strong (+++) respectively^20^.

### Statistical Analysis

The results are expressed as mean ± SD for continuous data and percentage (%) for categorical data. Mann–Whitney U test was used for the comparison of continuous variables, of which results are expressed as median. Pearson *χ*^2^ test or Fisher’s exact test was used for comparison of categorical variables. Multivariate logistic regression was performed to examine the associations between LNM and ultrasonographic characteristics as well as quantified level of *BRAF*^V600E^ mutation. The SPSS statistical software (version 22.0) was used for all analyses. A *P* value < 0.05 was considered to be significant.
